# Abnormal proplatelet formation and emperipolesis in cultured human megakaryocytes from gray platelet syndrome patients

**DOI:** 10.1038/srep23213

**Published:** 2016-03-18

**Authors:** Christian A. Di Buduo, Maria Adele Alberelli, Ana C. Glembostky, Gianmarco Podda, Paola R. Lev, Marco Cattaneo, Raffaele Landolfi, Paula G. Heller, Alessandra Balduini, Erica De Candia

**Affiliations:** 1 Department of Molecular Medicine, University of Pavia, Pavia, Italy; 2 Biotechnology Research Laboratories, Istituto di Ricovero e Cura a Carattere Scientifico (IRCCS) Policlinico San Matteo Foundation, Pavia, Italy; 3 Department of Internal Medicine, Policlinico Agostino Gemelli, Catholic University, Rome, Italy; 4 Hematology Research, Instituto de Investigaciones Médicas Alfredo Lanari, University of Buenos Aires, CONICET, Buenos Aires, Argentina; 5 Medicina III, Azienda Ospedaliera San Paolo, Dipartimento di Scienze della Salute, Università degli Studi di Milano, Milan, Italy; 6 Department of Biomedical Engineering, Tufts University, Medford, MA, USA

## Abstract

The Gray Platelet Syndrome (GPS) is a rare inherited bleeding disorder characterized by deficiency of platelet α-granules, macrothrombocytopenia and marrow fibrosis. The autosomal recessive form of GPS is linked to loss of function mutations in *NBEAL2*, which is predicted to regulate granule trafficking in megakaryocytes, the platelet progenitors. We report the first analysis of cultured megakaryocytes from GPS patients with *NBEAL2* mutations. Megakaryocytes cultured from peripheral blood or bone marrow hematopoietic progenitor cells from four patients were used to investigate megakaryopoiesis, megakaryocyte morphology and platelet formation. *In vitro* differentiation of megakaryocytes was normal, whereas we observed deficiency of megakaryocyte α-granule proteins and emperipolesis. Importantly, we first demonstrated that platelet formation by GPS megakaryocytes was severely affected, a defect which might be the major cause of thrombocytopenia in patients. These results demonstrate that cultured megakaryocytes from GPS patients provide a valuable model to understand the pathogenesis of GPS in humans.

The gray platelet syndrome (GPS) is a rare inherited platelet disorder characterized by mild to moderate bleeding manifestations, thrombocytopenia, large platelets, increased serum B_12_ levels, spleen enlargement and progressive myelofibrosis^1^,^2^,^3^. The distinctive feature of the disease is the deficiency of platelet α-granules responsible for the typical gray appearance of platelets on Wright-stained blood smears^1^. Platelet α-granules store cargo proteins that mediate platelet adhesion (e.g. von Willebrand Factor), hemostasis (e.g. factor V), inflammation (e.g. IL-1β, IL-8, platelet factor 4) and wound healing and angiogenesis (e.g. VEGF, FGF-2, PDGF)^4^.

Although the GPS displays an autosomal recessive inheritance in most cases^3^, sporadic families with autosomal dominant inheritance pattern have also been described^5^,^6^. Recently, by using a next generation sequencing approach, biallelic mutations in the neurobeachin-like 2 (*NBEAL2)* gene have been identified in autosomal recessive forms of GPS^7^,^8^,^9^. *NBEAL2* belongs to a family of proteins involved in the membrane dynamics and intracellular vesicle trafficking. One such protein, LYST, is mutated in Chediak-Higashi syndrome, which is characterized by defects in platelet granules and other lysosome-related organelles. The findings indicate that *NBEAL2* may be critical for the development of platelet α-granules. However, the mechanisms by which *NBEAL2* loss of function contributes to deficiency of platelet α-granules and their cargo proteins and to the macrothrombocytopenic state remain unknown.

Three different *Nbeal2* deficient (^−/−^) mouse strains have been generated^10^,^11^,^12^. All of them recapitulate the typical platelet phenotype observed in GPS patients. Mice have macrothrombocytopenia, deficiency of platelet α-granules and spleen enlargement. Myelofibrosis was demonstrated in older animals^12^. *Nbeal2*^−/−^ mice were used to extensively study the role of platelet α-granules constituents in hemostasis, thrombosis, thrombo-inflammatory disease states, megakaryocyte survival and development, platelet production, tissue reconstitution after injury, development of myelofibrosis and cancer metastasis propagation. However, some differences among the three *Nbeal2*^−/−^ mouse strains were reported with regard to megakaryocyte development and differentiation, proplatelet formation and α-granules content. Therefore, whether *Nbeal2* loss of function in mice affects megakaryopoiesis and/or proplatelet formation, and how it contributes to thrombocytopenia is unclear.

To investigate the impact of mutations in *NBEAL2* gene on human thrombopoiesis, we enrolled four GPS patients, whose clinical features and *NBEAL2* mutations have been previously described^13^,^14^,^15^. We obtained *in vitro* differentiated megakaryocytes from peripheral blood or bone marrow hematopoietic progenitor cells of the four GPS patients and five controls and evaluated megakaryocyte maturation and function and proplatelet formation.

## Results

### *NBEAL2* mutations do not affect megakaryocyte differentiation by human hematopoietic progenitors

**Figure 1:**
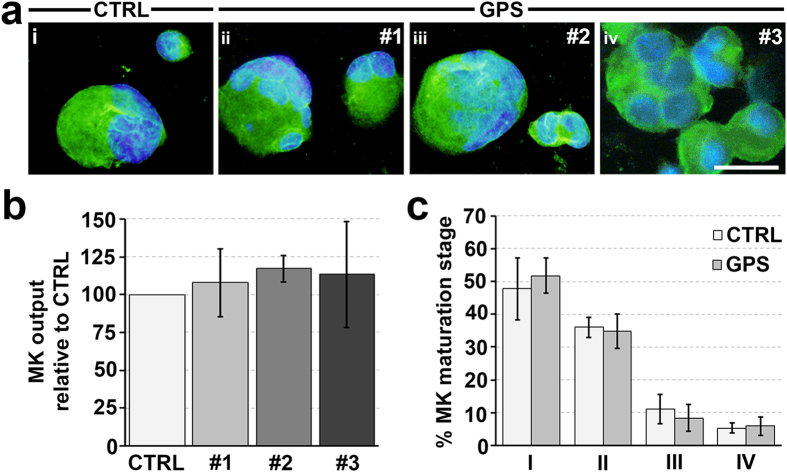
Normal differentiation of human megakaryocytes from patients with GPS. Hematopoietic progenitors from peripheral blood of healthy controls (CTRL) and patients with GPS were differentiated in culture into megakaryocytes in presence of TPO. (**a**) Representative immunofluorescence staining of plasma membrane CD61 in CTRL- (i) and GPS-derived megakaryocytes (ii–iv) (green = CD61; blue = nuclei; scale bar = 25 μm). (**b**) Statistical analysis of megakaryocyte (MK) output of GPS patients (#1, #2 and #3) relative to CTRL samples. Data are presented as mean ± SD (p = NS). (**c**) Statistical analysis of maturation stages in controls (CTRL)- and gray platelet syndrome patients- derived megakaryocytes (MK). Maturation stages were identified according to standard morphological criteria, as specified in the ‘Materials and Methods’ section. Data are presented as means ± SD of CTRL and all GPS patients GPS (p = NS). No differences were observed among patients.

We differentiated human megakaryocytes *in vitro* starting from peripheral blood or bone marrow hematopoietic progenitor cells of GPS patients with mutated *NBEAL2* and healthy controls. Hereinafter, the patient carrying the c.1253del, c.3584G>A and c.5720+1G>A (r.5720_5721ins148) mutations will be identified as #1; the patient carrying the c.5572C>T, c.6652G>T and c.7033C>T mutations will be identified as #2; the two patients carrying the c.2187C>A homozygous mutations will be identified as #3 (Table 1). After 14 days of culture, *in vitro* megakaryocyte differentiation and output of CD61^+^CD42b^+^ megakaryocytes were similar to those of healthy control samples (Fig. 1a,b). Further, maturation stages of CD61^+^ megakaryocytes, classified I to IV according to standard morphological criteria^16^, were also similar in GPS patients and controls (Fig. 1c). No differences were observed among patients #1, #2 and #3, thus indicating that all the analyzed *NBEAL2* mutations did not affect either the differentiation or the maturation of megakaryocytes. The results from both peripheral blood- and bone marrow-derived megakaryocytes were perfectively comparable (not shown).

### Reduced α-granule content in human megakaryocytes from GPS patients

**Figure 2:**
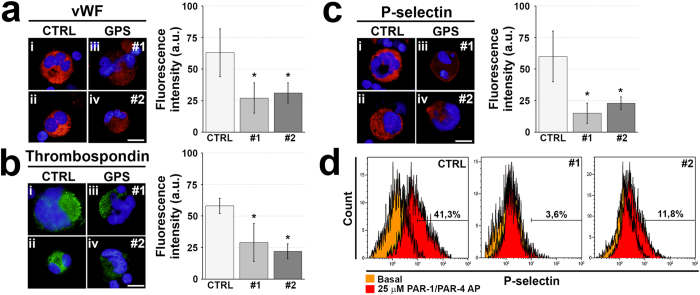
Lack of α-granule proteins in GPS cultured megakaryocytes. Fully differentiated megakaryocytes from healthy controls (CTRL) and patients with GPS were harvested and allowed to adhere onto Poly-L-Lysine coated cover-slips by centrifugation, fixed and analyzed. (**a**) Representative immunofluorescence staining of von Willebrand Factor (vWF) in CTRL- (i–ii) and GPS-derived megakaryocytes (iii–iv) (red = vWF; blue = nuclei; scale bar = 20 μm). Bars report the analysis of fluorescence intensity by Image J software (a.u. = arbitrary units). Data are presented as mean±SD (*p < 0.05). (**b**) Representative immunofluorescence staining of thrombospondin in CTRL- (i–ii) and GPS-derived megakaryocytes (iii–iv) (green = thrombospondin; blue = nuclei; scale bar = 20 μm). Bars report the analysis of fluorescence intensity by Image J software (a.u. = arbitrary units). Data are presented as mean ± SD (*p < 0.05). (**c**) Representative immunofluorescence staining of P-selectin in CTRL- (i–ii) and GPS-derived megakaryocytes (iii–iv) (red = P-selectin; blue = nuclei; scale bar = 20 μm). Bars report the analysis of fluorescence intensity by Image J software (a.u. =  arbitrary units). Data are presented as mean±SD (*p < 0.05). (**d**) Representative flow cytometry analysis of P-Selectin exposure by CTRL- and GPS-derived megakaryocytes in basal condition (orange) and upon stimulation with 25 μM PAR-1 and PAR-4 activating peptides (AP) (red). Bars highlight the increased percentage of P-Selectin^+^ megakaryocytes upon stimulation with respect to basal.

Despite normal differentiation, three of the most abundant proteins normally contained in α-granules, von Willebrand factor, thrombospondin and P-selectin, were markedly reduced in *in vitro* differentiated human megakaryocytes from both #1 and #2 GPS patients compared to controls (Fig. 2a–c). No differences were observed between patients, thus suggesting that the storage of these proteins was compromised in GPS megakaryocytes regardless of the type of mutation. Consistently, #1 and #2 GPS-derived megakaryocytes stimulated with protease activated receptors (PARs)-activating peptides (APs) exposed less P-selectin compared to control megakaryocytes (Fig. 2d). Interestingly, these results are in agreement with those we have recently observed in platelets from peripheral blood of patients #1 and #2^15^, and demonstrate that defective P-selectin mobilization is a distinctive feature that gray platelets inherit directly from their progenitors megakaryocytes before their release into bloodstream.

### Evidence of emperipolesis in human megakaryocytes from GPS patients

**Figure 3:**
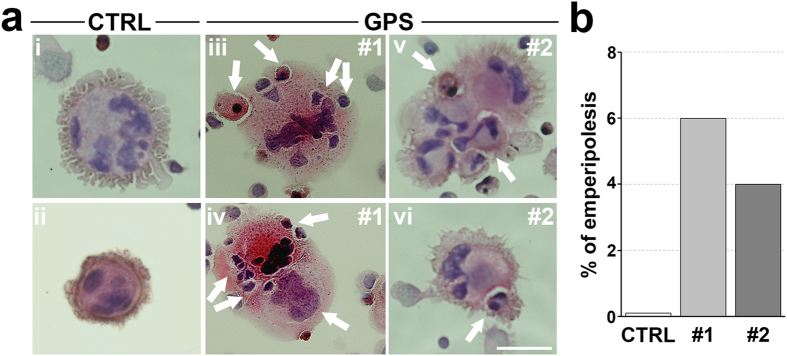
Emperipolesis in GPS cultured megakaryocytes: a distinctive feature of the disease is confirmed in *in vitro* model. (**a**) Representative hematoxylin and eosin staining of CTRL- (i–ii) and GPS-derived megakaryocytes (iii–vi). Arrows indicate intact cells that are going to and/or are already enclosed within megakaryocytes cytoplasm, thus indicating emperipolesis. (**b**) Percentage of emperipolesis in CTRL and GPS samples.

Bone marrow megakaryocytes from gray platelets syndrome patients display intact cells within their cytoplasm, as a consequence of a rare biological process that is named emperipolesis^6^,^15^,^17^. Emperipolesis, present in less than 2% of megakaryocytes in normal bone marrow biopsies, has been shown in 38% to 65% of megakaryocytes in GPS bone marrow biopsies^15^. However, whether this abnormality is directly dependent on *NBEAL2* mutation rather than a consequence of compromised bone marrow homeostasis, associated with the progressive myelofibrosis, has never been investigated. We found that several megakaryocytes from #1 and #2 GPS patients displayed the presence of intact cells within their cytoplasm, likely as a consequence of emperipolesis, while this abnormality was not observed in any of the mature megakaryocytes from control subjects (Fig. 3). The presence of cells other than megakaryocytes in our *in vitro* culture conditions is not surprising given the presence of a mixed population of cells in the culture media before megakaryocyte population is enriched through an albumin gradient^18^,^19^,^20^,^21^, which could grow and be engulfed within the megakaryocyte cytoplasm during the course of differentiation. To the best of our knowledge, none of the previously published studies of megakaryocytes from patients with other forms of thrombocytopenia or healthy subjects, grown under similar experimental conditions, revealed the presence of intact cells in cultured megakaryocytes^20^,^22^,^23^,^24^. Therefore, our study provides the first evidence that emperipolesis occurs in human GPS megakaryocytes as a consequence of an intrinsic cellular defect, rather than of abnormalities of the bone marrow microenvironment.

### Calcium signaling is maintained in human megakaryocytes with mutated NBEAL2 gene

**Figure 4:**
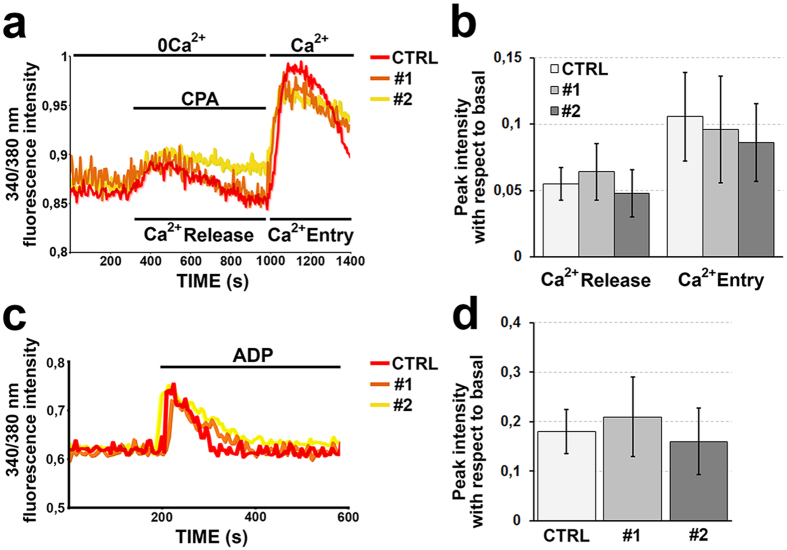
Calcium dynamics are maintained in gray platelet syndrome megakaryocytes. (**a**) Intracellular calcium (Ca^2+^) pools were depleted by exposing megakaryocytes to cycolpiazonic acid (CPA, 10 μM) in Ca^2+^-free (0Ca^2+^) solution. Re-addition of extracellular Ca^2+^ led to an increase in intracellular Ca^2+^ concentration which was indicative of activation of Store Operated Ca^2+^ Entry in both control (CTRL)- and #1 and #2 gray platelet syndrome (GPS)-derived megakaryocytes (red: CTRL; orange: #1 GPS; yellow: #2 GPS). (**b**) Statistical analysis of the peak amplitudes of both Ca^2+^ release and Ca^2+^ entry stimulated by CPA in CTRL- and #1and #2 GPS-derived megakaryocytes. Data are presented as means±SD (p = NS). (**c**) Adenosine diphosphate (ADP, 25 μM) evoked the activation of Ca^2+^ signaling in CTLR- and both #1 and #2 GPS-derived megakaryocytes (red: CTRL; orange: #1 GPS; yellow: #2 GPS). (**d**) Statistical evaluation of the effect of ADP treatment on the amplitudes of Ca^2+^ signaling in CTRL- and #1and #2 GPS-derived megakaryocytes. Data are presented as means±SD (p = NS).

We have recently demonstrated that constitutively released adenosine diphosphate (ADP) by human mature megakaryocytes promotes the activation of Store-Operated Calcium Entry (SOCE) which in turn is responsible for the regulation of platelet formation and interaction with the components of the extracellular matrix^19^,^25^. We explored SOCE functionality in GPS megakaryocytes from #1 and #2 patients by depleting the stores with cyclopiazonic acid (CPA, 10 μM). The treatment evoked a transient rise in intracellular calcium (Ca^2+^) concentration, because of passive emptying of endoplasmic reticulum, followed by influx of external Ca^2+^ through activated SOC channels (Fig. 4a). Analysis of the extent of both Ca^2+^ release and entry demonstrated no significant difference between control- and GPS-derived megakaryocytes (Fig. 4b). Consistent with this data, megakaryocytes from #1 and #2 GPS patients normally responded to ADP stimulation, with no significant differences in the extent of Ca^2+^ mobilization compared to control megakaryocytes (Fig. 4c,d). All together, these data indicate that Ca^2+^ homeostasis is normally maintained in GPS megakaryocytes.

### Human megakaryocytes from GPS patients show dramatically impaired proplatelet formation

**Figure 5:**
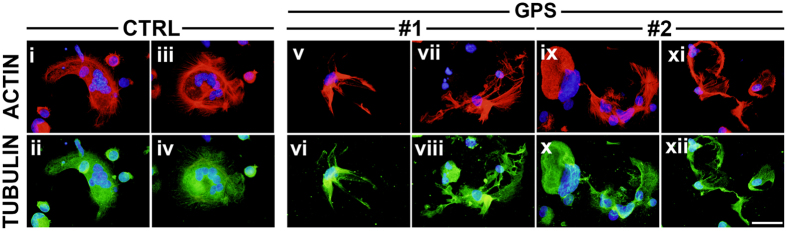
Anomalous cytoskeleton contractility of GPS megakaryocytes upon adhesion on type I collagen. Control (CTRL)- (i–iv) and GPS-derived megakaryocytes (v–xii) at the end of differentiation were plated on type I collagen-coated cover-slips, at 37 °C in a 5% CO_2_ atmosphere. After 16 hours adherent cells were fixed and stained for immunofluorescence analysis of the cytoskeleton components actin (red = TRTIC-phalloidin) and tubulin (green = α-tubulin). Nuclei were counterstained with Hoechst 33258 in blue (scale bar = 20 μm).

**Figure 6:**
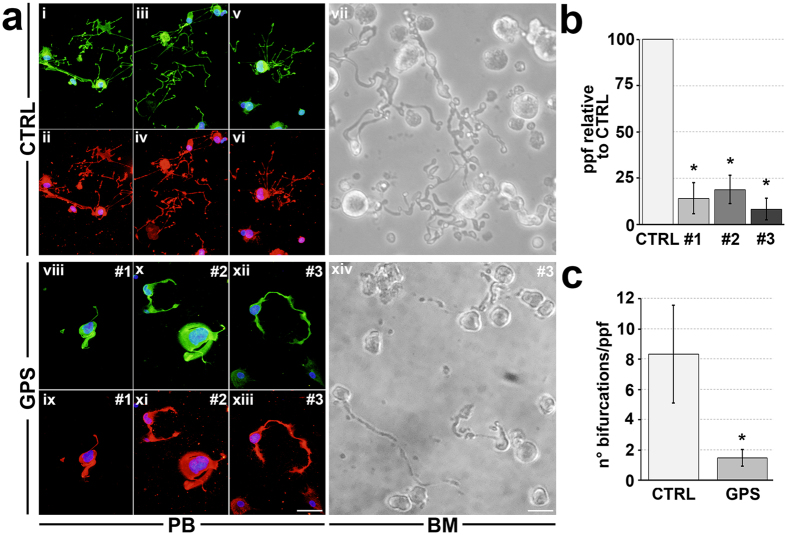
Aberrant proplatelet formation by human megakaryocytes from patients with GPS. (**a**) Representative analysis of proplatelet formation and structure from control (CTRL)- (i–vii) and GPS-derived megakaryocytes by immunofluorescence (peripheral blood sample i–vi and viii–xiii; green = α-tubulin; red = CD61; blue = nuclei; scale bar = 50 μm) and light microscopy (bone marrow samples vii, xiv; scale bar = 20 μm). Pictures clearly show defective proplatelet elongation in GPS patients. (**b**) The percentage of proplatelet forming-megakaryocytes (ppf) was calculated as the number of megakaryocytes displaying at least one filamentous pseudopod with respect to total number of round megakaryocytes per analyzed field, and normalized with respect to CTRL. Data are presented as means ± SD (*p < 0.001). (**c**) Analysis of the number of proplatelet bifurcations per proplatelet forming-megakaryocytes (ppf). Data are presented as mean ± SD (*p < 0.001).

**Figure 7:**
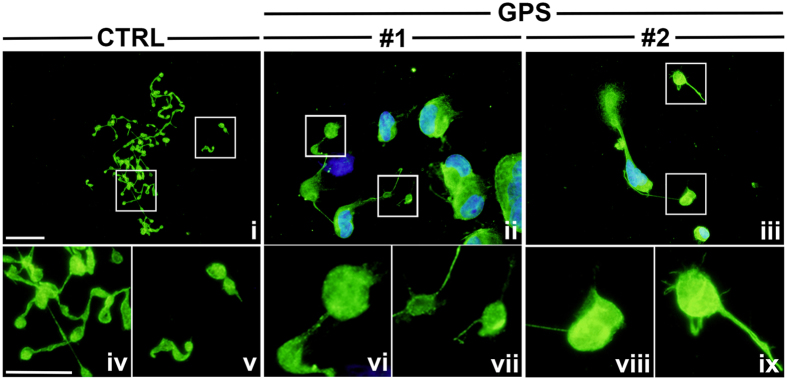
Giant proplatelet tips and released platelets from GPS megakaryocytes. Panel shows terminal proplatelet formation and platelet release by healthy controls (CTRL) (i) and GPS (ii,iii) megakaryocytes *in vitro* (green = α-tubulin; blue = nuclei; scale bar = 40 μm). Boxes highlight terminal proplatelet tips and platelet released in culture. Noteworthy, the size of both platelets tips and released platelets is markedly increased in GPS (vi,vii and viii,ix) than in CTRL (iv,v) samples (green = α-tubulin; scale bar = 20 μm).

An efficient Ca^2+^ signaling is fundamental in order to guarantee proper megakaryocyte interaction with extracellular matrix components of the bone marrow environment^25^, which is in turn relevant for the control of platelet release^26^,^27^. Therefore, based on the demonstration of a normal Ca^2+^ homeostasis in GPS megakaryocytes, we expected that they could normally interact with extracellular matrix components. Surprisingly, the analysis of GPS megakaryocyte interaction with type I collagen, an extracellular matrix component that promotes megakaryocyte spreading by supporting cytoskeleton contractility^27^, revealed abnormal actin stress fibers formation and microtubule assembly and, consequently, defective megakaryocyte spreading (Fig. 5). Similar results were observed in samples from #1 and #2 patients (Fig. 5v–xii). Further, the analysis of proplatelet formation on fibronectin^28^,^29^, revealed that megakaryocytes derived from either #1, #2 or #3 GPS patients displayed shorter proplatelet branches compared to control megakaryocytes (Fig. 6a). Similar results were obtained from both peripheral blood- (Fig. 6ai–vi,viii–xiii) and bone marrow-derived megakaryocytes (Fig. 6avii,xiv). The percentage of proplatelet forming megakaryocytes was markedly reduced in diseased megakaryocytes compared to controls, while no significant differences were observed among #1, #2 and #3 samples (Fig. 6b). The tips, terminal ends of proplatelet branches, are the site of the assembly of nascent platelets^30^. Consequently, the number of platelets that are formed depends on both the percentage of proplatelets and the number of their bifurcations in order to ensure the highest number of tips per single megakaryocyte. Proplatelets from GPS megakaryocytes displayed abnormal architecture with significant reduction in the absolute number of bifurcations compared to healthy controls (Fig. 6c). These results were consistent with median platelet number of GPS patients, which was significantly lower in #1, #2 and #3 patients (56 × 10^3^ platelets/μl, range: 30–65) than in healthy controls (294 × 10^3^ platelets/μl, range: 200–380). Moreover, we observed the presence of giant proplatelet tips and released platelets in cell cultures from #1 and #2 GPS patients (Fig. 7), consistent with the findings that these patients displayed a significant macrothrombocytopenia (mean platelet volume: 3.9 μm, range: 3.5–4.3) with respect to healthy controls (mean platelet volume: 2.4 μm, range: 2–2.7). All together these data suggest that altered megakaryocyte interaction with extracellular environment, together with a severe defect in proplatelet formation and branching, may be the major causes of reduced platelet count and increased platelet size that characterize GPS.

## Discussion

To the best of our knowledge, this the first report on megakaryopoiesis and proplatelet formation in cultured megakaryocytes from GPS patients carrying different autosomal recessive *NBEAL2* mutations (Table 1). The observed results were, therefore, independent of the mutations or of their location to a particular domain of the gene.

*NBEAL2* mutations did not affect either the differentiation or the maturation of human megakaryocytes. Similar results were obtained in different patients, using different sources (peripheral blood and bone marrow) of hematopoietic progenitor cells to differentiate along the megakaryocytic lineage in culture. Our results are in agreement with the clinical findings that, despite the presence of myelofibrosis, the number and the maturation of megakaryocytes are not abnormal in patients with GPS^2^. *Nbeal2*^−/−^ mouse models, to this regard, displayed different behaviors, which did not clarify the role of Nbeal2 in megakaryocyte differentiation^10^,^11^,^12^. In the bone marrow of *Nbeal2*^−/−^ mice, compared to control mice, the megakaryocyte number was increased in one strain^11^, slightly increased in older mice in another strain and similar to control mice in the third one^12^. Cultured megakaryocytes from bone marrow revealed normal differentiation and number of high ploidy megakaryocytes in one model^12^ and reduced ploidy together with megakaryocytes arrested in the earliest stage of differentiation in another model^10^. Our findings in GPS patients show that *NBEAL2* in humans does not affect the megakaryopoiesis, regardless of the type of mutations affecting the patients.

We also found a marked reduction of α-granule cargo and membrane proteins in mature megakaryocytes from #1 and #2 GPS patients compared to controls. These results are consistent with previous findings in the bone marrow biopsies of GPS patients^15^,^31^,^32^ and in the bone marrow of two *Nbeal2*^−/−^ mouse models^10^,^11^. Moreover, they are in agreement with the demonstration that *NBEAL2* is involved in the biogenesis/trafficking of granules in megakaryocytes^7^,^8^,^9^. Altogether these findings, although do not clarify whether the cause of reduced α-granule proteins is a hampered granule synthesis or a lack of retention of α-granules within the megakaryocytes, do confirm that the lack of α-granule proteins is present at the level of megakaryocytes (both culture-derived *in vitro* and in the bone marrow *in vivo*), and does not occur in platelets upon release of α-granules content into the circulation.

Emperipolesis was found in *in vitro* cultured GPS megakaryocytes from #1 and #2 patients. Emperipolesis, specifically the presence of leukocytes within the cytoplasm of megakaryocytes, has been already described as a prominent feature in bone marrow samples from GPS patients^17^, carrying both *NBEAL2*^15^ and *GFI1B* mutations^6^, and in all three *Nbeal2*^−/−^ mice^10^,^11^,^12^. These consistent findings suggest that this is a distinctive feature of GPS independent of the genotype and might be crucial for the development of the platelet phenotype. Our results on cultured megakaryocytes demonstrate that emperipolesis is intrinsic to these cells, independent of influence from microenvironment and/or other cells. Although the mechanism that favors emperipolesis in GPS is still far to be elucidated, some evidences suggest a mechanism similar to that described in primary myelofibrosis in humans, involving the mislocalization of P-selectin on megakaryocyte membrane favoring the interaction with leukocytes through the PSGL-1 countereceptor and their trapping within the megakaryocyte cytoplasm^33^,^34^. Also, a proinflammatory phenotype of *Nbeal2*^−/−^ megakaryocytes overproducing and releasing chemoattractans has been hypothesized as the cause of this phenomenon^12^. Whatever the mechanism involved in this phenomenon, Guerrero *et al*. demonstrated that engulfment of cells within the megakaryocytes does not affect their survival and apoptosis^12^.

Ca^2+^ homeostasis was normal in #1 and #2 GPS megakaryocytes. Conversely, we report for the first time a dramatic defect in proplatelet formation and megakaryocyte interaction with extracellular matrix. GPS megakaryocytes, from all studied mutations and from both peripheral blood and bone marrow samples, displayed shorter proplatelet branches, a significant reduction in the absolute number of their bifurcations and abnormal architecture of proplatelets when compared to control megakaryocytes. Moreover, the percentage of proplatelet forming megakaryocytes was markedly reduced compared to controls, independently of genotype, in agreement with results obtained in *Nbeal2*^−/−^ mice by Kahr *et al*.^10^. Unaltered proplatelet formation was reported in the other two *Nbeal2*^−/−^ mouse models^11^,^12^. We also found altered GPS megakaryocyte interaction with type I collagen, a component of bone marrow extracellular matrix responsible for control of proper platelet formation through regulation of cytoskeleton contractility. It is well known that the release of cellular fibronectin, stored in α-granules, by mature megakaryocytes adhering on type I collagen is the major determinant for the maintenance of the tight interaction between cells and the substrates, which in turn favors the cytoskeleton contractility^27^. Thus, impaired α-granule assembly may be the cause for the altered megakaryocyte contractility in GPS patients.

Defective proplatelet formation together with altered megakaryocyte interaction with extracellular environment may be the major causes of congenitally reduced platelet count in patients with GPS. Other factors partially contributing the worsening of macrothrombocytopenia with age are splenomegaly and myelofibrosis. However, spleen removal causes a mild increase of the platelet count^3^, thus suggesting that splenomegaly is not the major cause of thrombocytopenia. Myelofibrosis is progressive in GPS and can account for the progressive reduction of platelet count as patients become older, but not for thrombocytopenia during childhood, when myelofibrosis can be absent^3^. By showing *in vitro* reduced proplatelet formation in cultured megakaryocytes from GPS patients, we provide a new mechanism for the thrombocytopenia in these patients.

In conclusion, this is the first systematic analysis of human, cultured GPS megakaryocytes and represents a comparator to the extensive work done with the *Nbeal2*^−/−^ mice. Some differences and some similarities between *NBEAL2* deficient megakaryocytes from GPS patients and *Nbeal2* deficient megakaryocytes from mouse models are reported. We demonstrate that cultured human megakaryocytes from GPS patients with autosomal recessive mutation of *NBEAL2* undergo normal differentiation and maturation and display, instead, severely impairment of both proplatelet formation and interaction with extracellular matrix. These two defects might be the major causes for the thrombocytopenia. We provide first evidence that emperipolesis, a distinctive feature of GPS, is a megakaryocyte-dependent phenomenon. Decreased content of α-granule proteins was found in cultured megakaryocytes, confirming that this phenomenon occurs at the level of megakaryocytes and not in circulating platelets. Altogether these findings demonstrated that *in vitro* cultured megakaryocytes represent a valuable tool in understanding human GPS and it might of potential use to dissect the function of *NBEAL2* at the molecular and cellular level to determine which trafficking steps are affected in the disease.

## Materials and Methods

### Materials

Adenosine 5′-diphosphate (ADP), Hoechst 33258, Poly-L-lysine solution, paraformaldehyde (PFA), eosin-hematoxylin solution, Triton X-100, tetramethyrhodamine isothiocyanate (TRITC)-conjugated phalloidin and cyclopiazonic acid from *Penicillium cyclopium* (CPA) were from Sigma Aldrich (Milan, Italy). Fura-2 acetoxymethyl ester (Fura-2 AM) was from Molecular Probes Europe BV (Leiden, The Netherlands). Human fibronectin was from BD Bioscience (Milan, Italy). Type I collagen was purified as described previously^35^. The following antibodies were used: monoclonal anti-CD61 (clone SZ21) (Immunotech, Marseille, France); goat monoclonal anti-CD61 (clone C-20) and mouse monoclonal anti-P-Selectin (clone 1E3) (Santa Cruz Biotechnology, California, USA); mouse monoclonal anti-α-tubulin (clone DM1A) and mouse monoclonal anti-human P-Selectin-allophycocyanin (APC) (clone clone AK4) (Sigma Aldrich, Milan, Italy); rabbit polyclonal anti-von Willebrand Factor (Dako, Milan, Italy); mouse monoclonal anti-Thrombospondin (clone A6.1), mouse monoclonal anti-CD61-fluorescein isothiocyanate (FITC) (clone PM6/13) and mouse monoclonal anti-CD42b-phycoerythrin (PE) (clone HIP1) (Abcam, Cambridge, UK); anti-CD61-FITC and anti-CD42b-PE (BD Biosciences, San José, CA, USA); Alexa Fluor-conjugated antibodies (Invitrogen, Milan, Italy).

### Solutions

Physiological salt solution (PSS) had the following composition: NaCl 150 mM, KCl 6 mM, CaCl_2_ 1.5 mM, MgCl_2_ 1 mM, glucose 10 mM, Hepes 10 mM. In Ca^2+^-free solution (0Ca^2+^), Ca^2+^ was substituted with NaCl 2 mM and EGTA 0.5 mM was added. Solutions were titrated to pH 7.4 with NaOH.

### Patients

Clinical and genetic features of patients 1 and 2, from Italy and of patients 3A and 3B (brothers), from Argentina, have been already reported^13^,^14^,^15^. Briefly, at the time of this study patients 1, 2, 3A and 3B were, respectively, 23, 8, 68 and 73 year-old. All patients had similar degree of macrothrombocytopenia, as reported in the result section, whereas leukocyte and red blood cells counts were not affected. All the details regarding the type of *NBEAL2* mutations are reported in Table 1.

Blood (all patients) and bone marrow (patient 3A) samples were obtained with informed consent of the patients or of the parents in case of minor age. Four healthy volunteers were recruited as controls for peripheral blood samples. Bone marrow sample from one patient with Ewing sarcoma, who underwent bone marrow biopsy for staging of the disease and without bone marrow involvement, was used as a control for bone marrow sample. Cultures of peripheral blood and of bone marrow samples of patients and controls were run in parallel in each experiment. The study was performed in accordance with the principles of the Declaration of Helsinki. The study was approved by the ethical committees of Policlinico Agostino Gemelli, Rome, Italy and of Instituto de Investigaciones Médicas Alfredo Lanari, CONICET, Buenos Aires, Argentina, involved in the management of enrolled patients.

### Differentiation of human megakaryocytes and morphological analysis

CD45^+^ from peripheral blood or CD34^+^ from bone marrow samples were separated by immunomagnetic bead selection (Miltenyi Biotec, Bologna, Italy) and cultured in Stem Span medium supplemented with 10 ng/mL thrombopoietin (TPO), interleukin (IL)-6 and IL-11 at 37 °C in a 5% CO_2_ fully humidified atmosphere, as previously described^18^,^20^,^22^. At the end of the culture (14^th^ day) 150 × 10^3^ cells were collected, cytospun on glass coverslips and stained with a primary antibody against CD61 (1:100) to evaluate megakaryocyte differentiation. Nuclei were stained with Hoechst 33258 (1:10000). CD61^+^ megakaryocytes were assigned to distinct stages of maturity according to standard morphological criteria^16^, as previously described^20^,^21^,^36^. Specifically, in the early maturation stage (I) megakaryocytes present the lowest cytoplasmic/nuclear ratio, compact nucleus and small size. Successive stages (II, III) are identified by progressive cytoplasmic mass increase and highly lobulated nuclei. Fully differentiated megakaryocytes (stage IV) present the highest cytoplasmic/nuclear ratio. For each specimen, at least 100 megakaryocytes were evaluated.

For the analysis of the α-granules content, at day 14 of the culture megakaryocytes were harvested, seeded onto glass coverslips, previously coated with Poly-L-Lysine, and allowed to adhere by centrifugation at 240 × g. Cells were then fixed in 4% paraformaldehyde, permeabilized with 0.1% Triton X-100, and stained with anti-von Willebrand Factor (1:100), anti-thrombospondin (1:50) or anti–P-Selectin (1:100). Nuclei were stained with Hoechst 33258 (1:10000). The fluorescence intensity was quantified by Image J software. Values are reported as arbiter units (a.u.). For each specimen, at least 100 megakaryocytes were evaluated.

For the analysis of emperipolesis, after fixation, samples were stained with hematoxylin and eosin. For each specimen, at least 200 megakaryocytes were evaluated. Results are expressed as percentage of total cells analyzed.

For all experiments the coverslips were mounted onto glass slides with ProLong Gold antifade reagent (Invitrogen, Milan, Italy) and images acquired by Olympus BX51 microscope (Olympus, Deutschland GmbH, Hamburg, Germany).

### Flow cytometry analysis

200 × 10^3^ cells were collected at the end of the culture (14^th^ day) and centrifuged at 250xg for 7 minutes. Cells were then suspended in phosphate buffer saline (PBS) and stained with a FITC-conjugated antibody against human CD61 and a PE-conjugated antibody against human CD42b, at room temperature, in the dark for 30 minutes. The number of CD61^+^CD42b^+^ megakaryocytes (megakaryocyte output) from GPS patients was calculated and evaluated with respect to those of healthy controls.

For analysis of P-Selectin exposure samples were stimulated with 25 μM of both PAR-1 and PAR-4 activating peptides for 20 minutes, at 37 °C, in presence of 2 μM CaCl_2_ and 2 μM MgCl_2_. Cells were then stained with APC-conjugated antibody against human P-Selectin antibody for additional 15 minutes. In all described experiments, after incubation, samples were immediately analyzed by a Beckman Coulter Navios flow cytometer. Non-stained samples were used to set the correct analytical gating. Off-line data analysis was performed using Beckman Coulter Navios software package.

### Evaluation of cell adhesion and proplatelet formation on fibronectin and type I collagen

In order to analyze megakaryocyte adhesion and proplatelet formation onto different extracellular matrix components, 12 mm glass cover-slips were coated with 25 μg/ml fibronectin or 25 μg/ml type I collagen, overnight at 4 °C, as previously described. At the end of the culture megakaryocyte population was enriched through a BSA gradient as previously described^20^. 1 × 10^5^ megakaryocytes were harvested and allowed to adhere at 37 °C and 5% CO_2_. After 16 hours, adhering cells were visualized either by phase contrast images by an Olympus IX53 microscope (Olympus, Deutschland GmbH, Hamburg, Germany) or fixed in 4% PFA, permeabilized with 0.1% Triton X-100, and stained for immunofluorescence evaluation by an Olympus BX51 microscope (Olympus, Deutschland GmbH, Hamburg, Germany). Briefly, for immunofluorescence staining samples were stained with anti-α-tubulin antibody (1:700), TRITC-conjugated phalloidin (1:5000) and/or CD61 (1:100), as previously described^25^,^27^. Nuclei were stained with Hoechst 33258 (1:10000). The cover-slips were mounted onto glass slides with ProLong Gold antifade reagent (Invitrogen, Milan, Italy). At least 50 fields per sample were analyzed. Cytoskeleton reorganization was recognized in cells displaying microtubule and stress fibers assembly. Proplatelet forming-megakaryocytes were identified as cells displaying long filamentous structure ending with platelet-sized tips. The percentage of proplatelets from GPS patients was compared with respect to those of healthy controls.

### Measurements of intracellular calcium concentration

The analysis of Ca^2+^ flows was performed as previously described^25^,^37^. 12 mm glass coverslips were coated with 25 μg/ml fibronectin, overnight at 4 °C. Mature megakaryocytes were plated onto substrate-coated cover-slips in 24-wells plates (1 × 10^5^ cells/well). After 60 minutes at 37 °C and 5% CO_2_, megakaryocytes were loaded with 4 μM fura-2 AM in PSS. After 30 minutes, the coverslip was fixed to the bottom of a Petri dish and the cells were observed using an upright epifluorescence Axiolab microscope (Carl Zeiss). Custom software, working in the LINUX environment, was used to drive the camera (Extended-ISIS Camera; Photonic Science) and the filter wheel and to measure and plot on-line the fluorescence from single cells enclosed in rectangular regions of interest (ROI). Intracellular Ca^2+^ concentration ([Ca^2+^]_i_) was monitored by measuring, for each ROI, the ratio of the mean fluorescence emitted at 510 nm when exciting alternatively at 340 and 380 nm (shortly termed “RATIO”). An increase in [Ca^2+^]_i_ causes an increase in the ratio. The experiments were performed at room temperature.

### Statistic

Megakaryocyte cultures were performed three times from peripheral blood of patient 1, two times from peripheral blood of patient 2, two times from peripheral blood and one time from bone marrow of patient 3A, one time from peripheral blood of patient 3B and each experiment was independently replicated at least 3 times, unless specified otherwise. Control samples from healthy volunteers (peripheral blood samples) and from one patient with Ewing sarcoma and no bone marrow involvement (bone marrow sample) were always performed in parallel. For all the experiments values are expressed as mean ± SD of all independent experiments. Student’s t-test was performed for statistical analysis. Values of at least p < 0.05 were considered statistically significant.

## Additional Information

**How to cite this article**: Di Buduo, C. A. *et al*. Abnormal proplatelet formation and emperipolesis in cultured human megakaryocytes from gray platelet syndrome patients. *Sci. Rep*. **6**, 23213; doi: 10.1038/srep23213 (2016).

## Figures and Tables

**Table 1:** Mutations in *NBEAL2* previously identified in three unrelated families and four probands.

Patients	cDNA (RNA for spicing mutations)		Exon/Intron	Protein	Type of mutation	Transmission
Patient 1 Rome (Italy)	c.1253del	#1	12	p.His418Leufs*54	Frameshift	Maternal
	c.3584G>A		25	p.Arg1195AGln	Missense	Paternal
	c.5720+1G>A (r.5720_5721ins148)		i35	p.Met1908*	Splice-site Frameshift	Paternal
Patient 2 Milan (Italy)	c.5572C>T	#2	34	p.Arg1858*	Nonsense	Paternal
	c.6652G>T		41	p.Glu2218*	Nonsense	Paternal
	c.7033C>T		45	p.Arg2345Trp	Missense	Maternal
Patients 3A and 3B Buenos Aires (Argentina)**	c.2187C>A	#3	16	p.Tyr729*	Nonsense	Maternal and Paternal

^**^Family from Buenos Aires had two affected brothers with homozygous *NBEAL2* mutation. Clinical and laboratory features of the probands have been detailed elsewhere^14^.
