# Corrigendum: Abnormal proplatelet formation and emperipolesis in cultured human megakaryocytes from gray platelet syndrome patients

**DOI:** 10.1038/srep25027

**Published:** 2016-04-29

**Authors:** Christian A. Di Buduo, Maria Adele Alberelli, Ana C. Glembotsky, Gianmarco Podda, Paola R. Lev, Marco Cattaneo, Raffaele Landolfi, Paula G. Heller, Alessandra Balduini, Erica De Candia

Scientific Reports 6: Article number: 23213; 10.1038/srep23213 published online: 03182016; updated: 04292016.

The original version of this Article contained a typographical error in the spelling of the author Ana C. Glembotsky, which was incorrectly given as Ana C. Glembostky. This has now been corrected in the PDF and HTML versions of the Article.

